# Decontamination of radioactive cesium ions using ordered mesoporous monetite

**DOI:** 10.1039/c8ra02707b

**Published:** 2018-05-23

**Authors:** Ali F. Tag El-Din, Emad A. Elshehy, Mahmoud O. Abd El-Magied, Asem A. Atia, Mohamed E. El-Khouly

**Affiliations:** Department of Chemistry, Faculty of Science, Kafrelsheikh University Kafr El-Sheikh 33516 Egypt mohamedelkhouly@yahoo.com; Nuclear Materials Authority P. O. Box 530, El Maadi Cairo Egypt; Department of Chemistry, Faculty of Science, Menoufia University Menoufia Egypt

## Abstract

We report herein the fabrication of an environmentally friendly, low-cost and efficient nanostructured mesoporous monetite plate-like mineral (CaHPO_4_) as an adsorbent for removal of radioactive cesium ions from aqueous solutions. The phase and textural features of the synthesized mesoporous monetite were well characterized by XRD, FTIR, SEM, HRTEM, DLS, TGA/TDA, and N_2_ adsorption/desorption techniques. The results indicate that the cesium ions were effectively adsorbed by the mesoporous monetite ion-exchanger (MMT-IEX) above pH 9.0. Different kinetic and isotherm models were applied to characterize the cesium adsorption process. The fabricated monetite exhibited a monolayer adsorption capacity up to 60.33 mg g^−1^ at pH of 9.5. The collected data revealed the higher ability of CaHPO_4_ for the removal of Cs(i) from aqueous media in an efficient way.

## Abstract 2

This study involves the identification of environmentally friendly, low-cost and efficient nanostructured mesoporous monetite plate like mineral as an adsorbent for removal of cesium from aqueous solutions.

## Graphical abstract

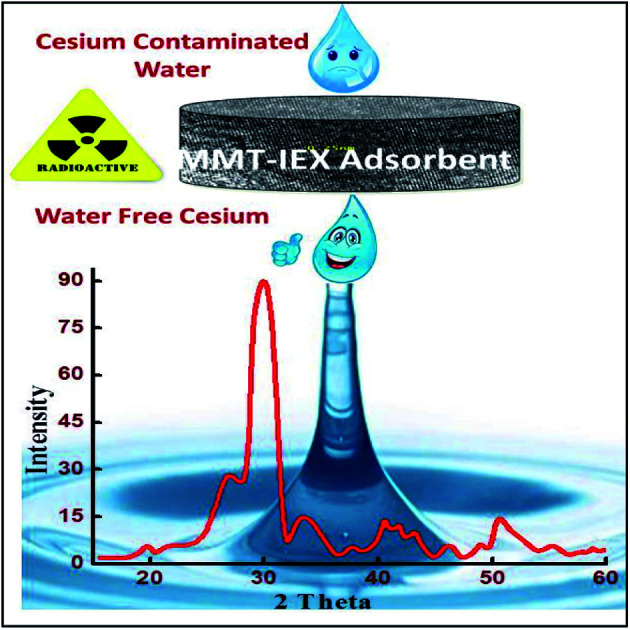

## 1. Introduction

Cesium (Cs) is an alkali metal with one naturally occurring isotope (^133^Cs) which is ubiquitous in the environment with concentrations in the Earth's crust ranging between 0.3 and 25 parts per million. As is well known, cesium isotopes are produced as byproducts of nuclear fission processes in nuclear reactors, nuclear weapons tests, and some nuclear accidents. These nuclear evolutions have resulted in high release of Cs isotopes into the aquatic environment. Among them, ^134^Cs, ^135^Cs, and ^137^Cs are of particular danger due to their high environmental mobility.^1–5^ The transport of radionuclides through aquatic systems is partially dependent on the physical and chemical properties of the nuclide and the water body.^1–3^ From a chemical point of view, Cs is a chemical analog to potassium, being a monovalent cation it would also tend to sorb to most negatively charged surfaces.

In the last decade, there has been considerable interest in sequestering of cesium ions from the aquatic environment. The applied technologies for the separation of cesium include: solvent extraction,^6^ precipitation,^4^ ion exchange,^5^ membranes,^7^ and adsorption.^8–10^ Among them, the ion exchange method has attracted much interest in recent years due to its rapid separation, high thermal and radiation stabilities of the adsorbents, and reduced amount of waste.

Various inorganic adsorbent materials and insoluble salts have been extensively utilized in cesium removal including illite, montmorillonite, kaolinite,^11^ nano-zeolite,^12^ zinc hexacyanoferrate,^13,14^ copper ferrocyanide,^15^ ammonium molybdophosphates,^1^ metal oxides,^16,17^ magnesium phosphate,^7^ tungstates,^18^ titanium and zirconium phosphate.^19^ Recently, calcium phosphate (CaP) materials, with different calcium-to-phosphate molar ratios, have attracted much attention for removing the radioactive species from contaminated media. Calcium based materials for removal of radioactive species has been focused on hydroxyapatite (HAP) and tri-calcium phosphate (TCP). Compared with them, dicalcium phosphate cements such as monetite (CaHPO_4_) are non-toxic and low cost. These advantages render it as promising materials in various applications such as: drug delivery, orthopedics, cancer therapy, biosensors, biological matrixes, and water treatment.^20–23^

The aim of the present study is to design simple and cost effective technique for the synthesis of ordered nanostructured mesoporous calcium hydrogen phosphate (*i.e.* monetite (MMT-IEX)) sorbent by a sol–gel method for cesium ion removal in an efficient way. The textural and structural characteristics of MMT-IEX were examined using different analytical and spectroscopic techniques. The effect of pH, eq. time, and cesium ion concentrations on the adsorption removal efficiency were clarified. The obtained adsorption data was applied to different isotherm models, kinetics and thermodynamic studies.

## 2. Materials and methods

### 2.1. Materials

Cesium nitrate solution (1000 mg L^−1^ in 0.05 M HNO_3_) was purchased from Merck Company. Calcium nitrate tetrahydrate (Ca(NO_3_)_2_·4H_2_O), ammonium hydroxide (NH_4_OH), cellulose acetate, ammonium dihydrogen phosphate (NH_4_H_2_PO_4_), phosphoric acid (H_3_PO_4_), ethanol and dodecylamine hydrochloride were ADWIC products, Egypt. The solution was further diluted to the required concentrations (1–150 mg L^−1^) with deionized water. The solution's pH was adjusted by 0.01 M nitric acid or 0.01 M sodium hydroxide solutions.

### 2.2. Fabrication of ordered nanostructured mesoporous monetite

Monetite meso-sorbent was synthesized using the conventional sol–gel method through three consecutive steps. Firstly, calcium nitrate (6.5 g) was dissolved in 9 mL ammonia solution and then 0.9 g dodecylamine hydrochloride was added to the solution with stirring. 2.3 g of NH_4_H_2_PO_4_ was dissolved in 10 mL H_2_O and then added to the former solution. Secondly, a 0.6 g cellulose acetate dissolved in water/ethanol solution (1 : 1) was subsequently added vigorous stirring to the former solution. The mixture was stirred for 3 h (at 70 °C; pH = 7.8) after which 5 mL H_3_PO_4_ (85%) was added dropwise with stirring. The reaction was performed at 70 °C for 6 h stirring until the gelatinous white precipitate (monetite-CA) was obtained. The gelatinous precipitate was kept with mother liquor for 24 h at room temperature and then the resulting adsorbent was filtrated and washed several times to remove remaining agents. Finally, the as-made MT-CA-DA was dried at 75 °C for 8 h and carefully heated in air by heating rate 25 °C min^−1^ from room temperature to 500 °C for 3 hours. The obtained material (MMT-IEX) was characterized and further used for the cesium ions removal process.

### 2.3. Materials characterization

Surface functional groups of the fabricated monetite were identified using Fourier transform infrared spectrometer (FTIR, 640-MSA, Thermo Electronics Co). The phase and mineral composition of the fabricated TM-MS was investigated by X-ray diffraction spectroscopy with a Philips X-ray generator model PW 3710 = 31. Thermo-gravimetric analysis (TGA) was carried out in a nitrogen atmosphere using Shimadzu DT = TG-50 with a heating rate of 10 °C min^−1^. The particle size distribution and zeta potential were measured using a Nano Series Zeta Sizer (Malvern; Worcestershire, UK). The particle size distribution and hydrodynamic diameter of the fabricated MMT-IEX were evaluated through dynamic light scattering (DLS). The sample (0.5 mg mL^−1^) was suspended in DIW and ultrasound-irradiated for 5 min. The cesium and conjugated ions concentrations were analyzed using inductively coupled plasma-optical emission spectrometer (ICP-OES, 720-ICP-OES, Agilent Technologies).

Pore-size distribution and Brunauer–Emmett–Teller (BET) specific surface area were calculated using the nitrogen adsorption/desorption method at 77 K (using a BELSORP MIN-II analyzer, JP. BEL Co. Ltd). Morphological characteristics of the adsorbent were examined using high-resolution field-emission scanning electron microscopy (ESEM) EXL 130 attached by energy dispersive spectrometry (EDX) unit system. High-resolution transmission electron microscopy (HRTEM), scanning electron microscopy (SEM), and elemental mapping of MMT-IEX product were performed using an energy-filtered TEM (EFTEM). The HRTEM, STEM, and STEM-EDS were operated at a camera length of 20 and a spot size of 1 nm. STEM and STEM-EDS were carried out at a camera length of 40 cm and a spot size of 0.7 nm. For characterization, the MMT-IEX was dispersed in ethanol solution using an ultrasonic cleaner and then dropped on a copper grid.

### 2.4. Batch studies for Cs(i) ions adsorption on MMT-IEX

During the adsorption process, the different parameters affected the characteristics of removal Cs(i) ions from aqueous solutions in terms of pH, time, concentration and metal ion diffusion were studied. In a typical Cs(i) ions-adsorption experiments, the MMT-IEX (30 mg) were immersed in a 20 mL of Cs(i) ions solution (150 mg L^−1^) and adjusted to the desired pH (1–11). The solutions contain MMT-IEX were shaken at 300 rpm for 90 min. After the batch contacts, the adsorbent was separated by filtration through a 25 mm Whatman filter paper and the remaining Cs ions concentration was determined by ICP-OES. The uptake of cesium ions *q*_e_ (mg g^−1^) is conducted according to eqn (1):^7^

1 *q*_e_ = (*C*_i_ − *C*_e_)*V/m*

where *q*_e_ is the amount of Cs(i) ions (mg) absorbed per one gram of MMT-IEX, *V* is the solution volume (L), *m* is the mass of the MMT-IEX (g), *C*_i_ and *C*_e_ are the initial and equilibrium concentrations of the cesium ion (mg L^−1^), respectively. After determining the pH values of the MMT-IEX, other experiments studies were conducted according to the specific pH value. In adsorption isotherm experiments, MMT-IEX (30 mg) was immersed in a 20 mL of different cesium ions concentrations at specific pH values with stirring for 90 min at room temperature.

## 3. Results and discussions

### 3.1. Characterization of the fabricated monetite

**Fig. 1:**
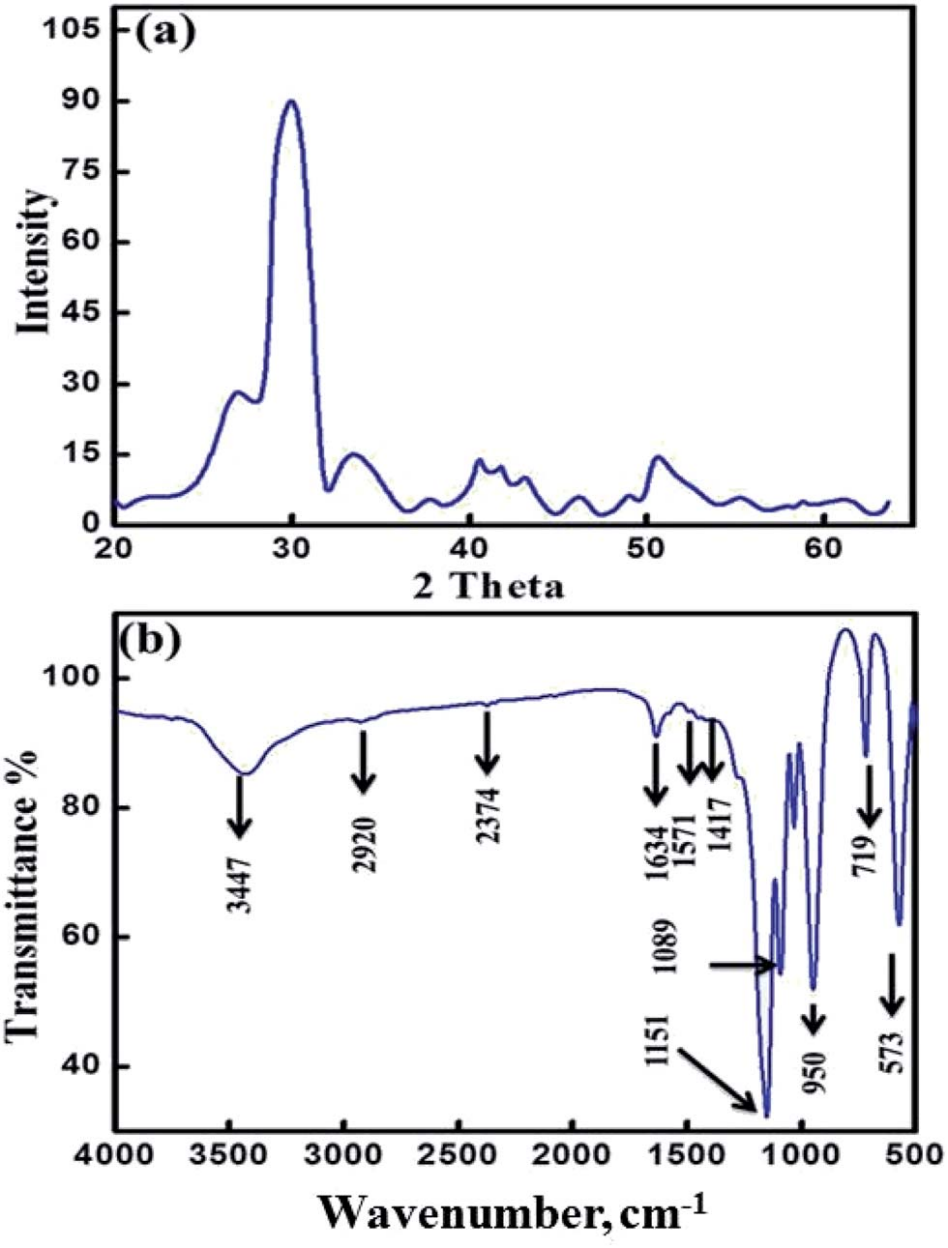
(a) X-ray diffraction pattern for monetite (CaHPO_4_) sample used in this study; Bragg reflections showed correspond to monetite as detailed in ICDD card number 04-012-8346. (b) FTIR spectra of the mesoporous monetite (MTT-IEX) sample nanoparticles.

The monetite, CaHPO_4_, crystallizes in a triclinic unit cell *a* = 6.9160 Å, *b* = 6.9160 Å, *c* = 6.9460 Å, *a* = 96.81, *b* = 103.82°, and *c* = 88.34° with *Z* = 4. Its structure consists of CaHPO_4_ chains bonded together by Ca–O bonds and three types of hydrogen bonds.^20–25^ Two distinct sets of pairs of PO_4_ units are found in each primitive cell with the *P*1 space group centered on a symmetric hydrogen bond and the other proton disordered over two centrosymmetrically related positions.^24,25^ The monetite (CaHPO_4_) was prepared by calcination of as-synthesized MT-CA/DA at 500 °C for 3 h due to template elimination (cellulose acetate and dodecylamine). Fig. 1a shows the XRD patterns of the mesoporous crystalline monetite structure.^20–24^ Further, the X-ray diffraction pattern exhibited two intense peaks at 2*θ* of 26.5° and 30.26° correspond to the calcium hydrogen phosphate. FTIR spectrum of MMT-IEX showed sharp three bands at 1152, 1089 and 950 cm^−1^ that attributed to P–O stretching vibration of PO_4_.^24,25^ The bands at 950 and 719 cm^−1^ are associated with asymmetric and symmetric stretching of P–O–P bonds, respectively. The intense bands at 2916, 2360, 1417 and 891 cm^−1^ are due to P–O(H) stretching and bending modes of the monetite structure. The observed stretching *ν*(OH) bands at different IR spectrum seem to be dependent on the three types of hydrogen bonds. Moreover, the absorption band at 573 cm^−1^ refers to stretching vibration of the O–P–O(H) bond. No OH-specific bands were found at 1571 and 631 cm^−1^, indicating that no hydroxyapatite appeared in the finally hardened products and the material is calcium hydrogen phosphate anhydrous.^25^

**Fig. 2:**
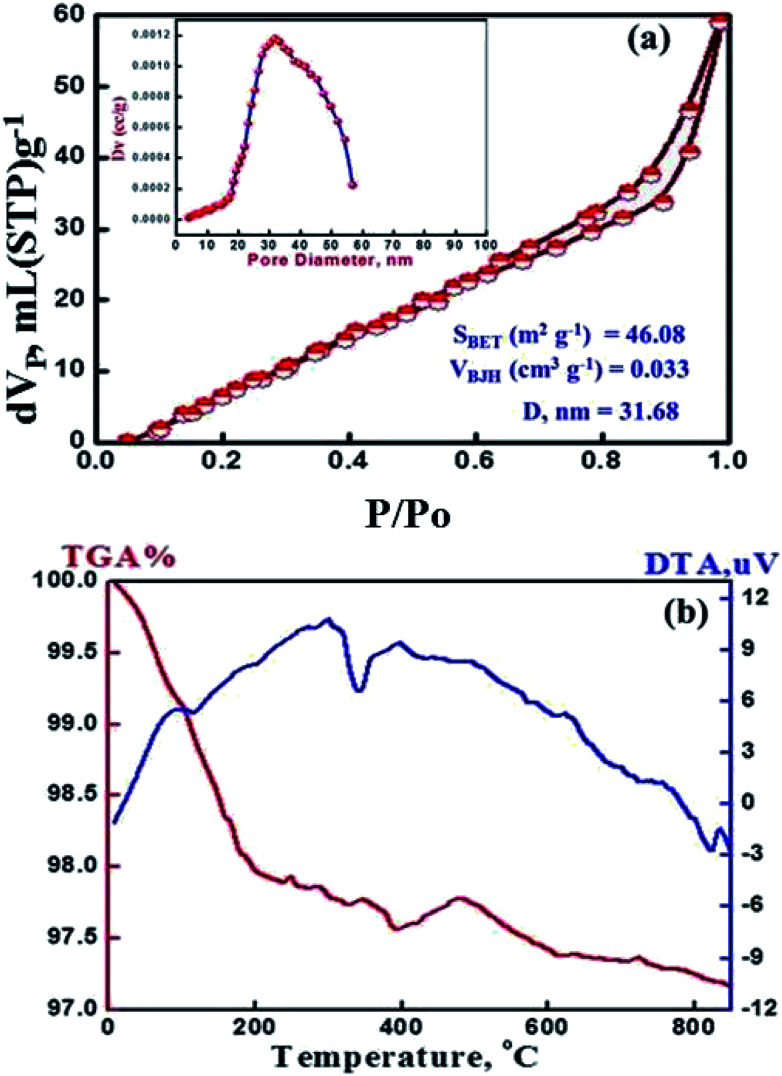
(a) N_2_ adsorption–desorption isotherm and pore size distribution plot (inset). (b) TGA/DTA of the mesoporous MMT-IEX nanoparticles.

The N_2_ isotherm shows a typical type IV adsorption behavior, with a well-known sharp adsorption/desorption inflection and the hysteresis loops shift to higher relative pressure (*P*/*P*_0_), implying an increase of the pore diameter and the formation mesoporous sorbent. Moreover, the isothermal shape and capillary evaporation indicate the formation of uniformly sized ordered mesopores with pore openings, surface area and pore volume of 31.68 nm, 47.0 m^2^ g^−1^ and 0.0333 cm^3^ g^−1^, respectively. Our results show that the assisted cellulose acetate/dodecylamine hydrochloride can be used to fabricate uniformly mesopores of MMT-IEX. TGA curves display weight losses of 4% for the calcined MMT-IEX in the temperature range 455–470 °C. These weight losses correspond to physisorbed water (Fig. 2b). The DTA curve of MMT-IEX shows two well-pronounced peaks, one endothermic peak in the temperature around 370 °C. This endothermic peak is due to desorption of water and CO_2_. The exothermic peak indicates that the reformation of the crystalline phase of monetite occurs in temperature at 986 °C.

**Fig. 3:**
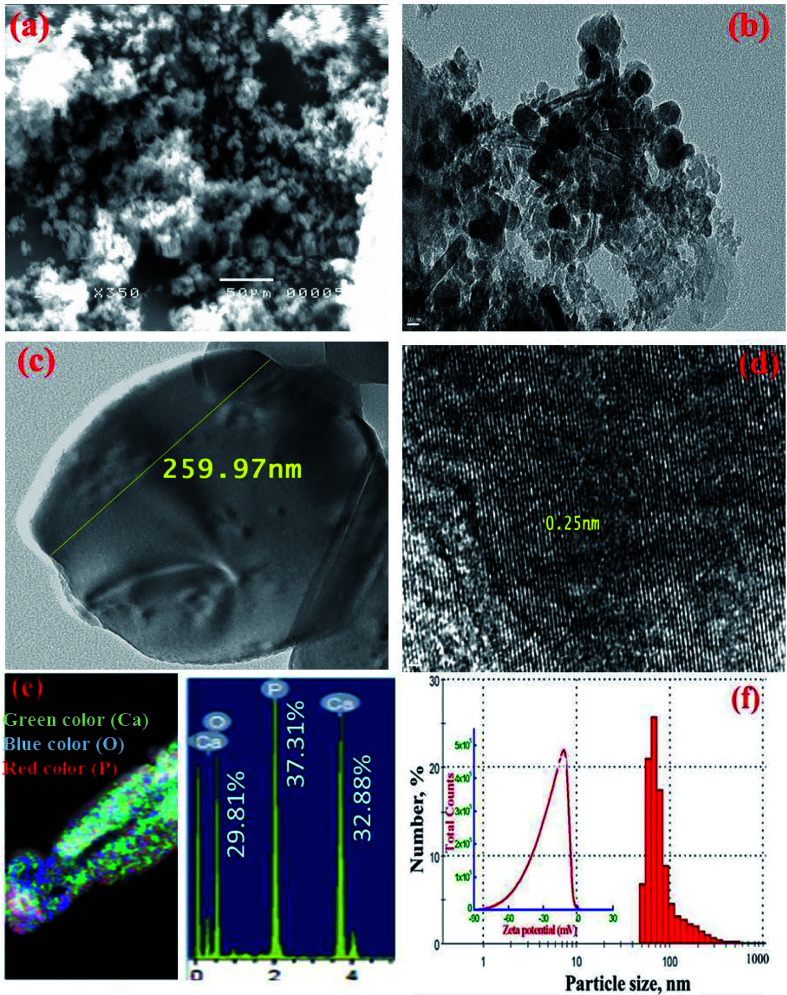
(a) Representative SEM, (b and c) TEM image for monetite nano-plates with different magnifications, (d) HRTEM shows evidence of the formation of ordered mesoporous structures, (e) STEM images of calcium (green), oxygen (blue) and phosphorous (red) and EDS analysis results, and the calculated values of the atomic abundance of the species present, and (f) particle size distribution and zeta potential (inset) of mesoporous MMT-IEX sorbent.

SEM and TEM images of MMT-IEX sorbent (Fig. 3a–c) show the typical morphology expected for monetite with a rectangular, plate-like crystals. The HRTEM image clearly shows the existence of a crystalline structure and confirming that the sample has an ordered mesoporous structure (Fig. 3d). STEM mappings combined with energy dispersive X-ray spectroscopy (STEM-EDS) analysis were performed to characterize the composition and atomic distribution of fabricated MMT-IEX nanospheres (Fig. 3e). STEM-EDS mapping of the MMT-IEX indicated the presence of O (29.81%), P (37.31%) and Ca (32.88%) in the composition domains of the monetite mesostructure (Fig. 3e). The dynamic light scattering (DLS) analysis of the monetite exhibits a uniform size in the range of 50–90 nm in diameter, suggesting the formation of monetite nanoparticles (Fig. 3f).

### 3.2. Adsorption assays of cesium ions on MMT-IEX

#### 3.2.1. Effect of pH

**Fig. 4:**
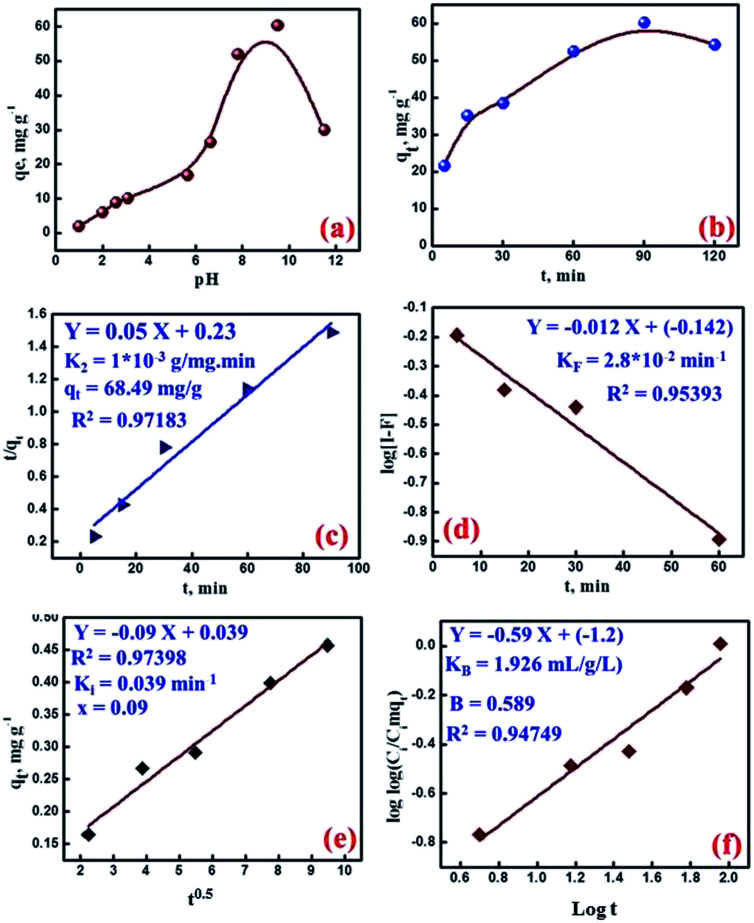
(a) Effect of pH on the adsorption of Cs(i) ions on CaP-MS; initial Cs(i) concentration 150 mg L^−1^, MMT-IEX weight 30 mg, solution volume 20 mL, contact time 90 min and 25 °C. (b) Effect of contact time on the adsorption of Cs(i) ions on MMT-IEX from a single ion solution; pH 9.5 initial Cs(i) concentration 150 mg L^−1^, MMT-IEX weight 30 mg, solution volume 20 mL at 25 °C. Kinetic profile of the cesium adsorption on MMT-IEX with different models. (c) The pseudo-second-order, (d) Weber–Morris, (e) McKay, and (f) Bangham plots for the adsorption cesium on MMT-IEX sorbent.

pH factor plays an important role in controlling the sorption process of the cesium ions. Moreover, it affects both the electrical charge on the surface of the MMT-IEX and the speciation of cesium ions in the solutions. Furthermore, zeta potential depends not only on the particle's surface properties but also the nature of the solution (*e.g.* ionic strength, pH, *etc.*). The surface zeta potential of MMT-IEX was determined to be −22.1 mV (Fig. 3f). The negative value is due to the deprotonated –OH group of the phosphate moiety that can explain the pH variations of uptake of cesium ions from solution by MMT-IEX. The effect of initial pH solution on the uptake of Cs(i) ions by MMT-IEX was evaluated over the pH ranges from 1.0 to 11.0 as shown in Fig. 4a. In ion exchange adsorbent (eqn (2)), Cs(i) uptake at acidic pH values was suppressed due to the excessive hydronium ions (H_3_O^+^) near the surface could produce a repulsive force to hinder the approach of the Cs(i) ions to the sorbent surface.^7^

2 MMT-IEX–OH + Cs^+^ → MMT-IEX–OCs + H^+^

At higher pH ranges, the surface sorption sites were deprotonated leading to the increase of electrostatic interaction to take up Cs(i) with higher efficiency with a maximum adsorption between pH 7.5 and 9.5. It is worth mentioning that the acidity of the medium decreased due to the release of H^+^ ions as a result of the ion exchange of the surface protons by Cs(i) ions through the ion exchange mechanism.

#### 3.2.2. Effect of equilibrium time

The time dependence of Cs(i) adsorption on the MMT-IEX was carried out mainly for two purposes; to reveal the applicability of MMT-IEX for the Cs(i) removal and to predict the adsorption mechanism. Adsorption of Cs(i) ions on MMT-IEX was studied within a time of 2 h at 25 °C and pH 9.5 and the results obtained are shown in Fig. 4b. Within 15 min, the uptake of Cs(i) represents 58.34% from the uptake capacity at the plateau. After that, the rate of Cs(i) adsorption slowed and reached the plateau region when the surface becomes saturated (60.33 mg g^−1^) within 90 min from a synthetic water sample containing 150 mg L^−1^ of cesium ions. The reason could be attributed to the porous structural features of MMT-IEX which facilitating the sorption of Cs(i) as well as surface hydrophilicity could be a factor responsible for surface active sites functional group interaction, which is entropically favorable. After 90 min, the Cs(i) adsorption was decreased dramatically by increasing the time due to increasing of [H^+^] in a solution that drives the reaction to auto-desorption process.

The kinetic studies are of great importance for both gaining insights on the physical chemistry of the sorption processes and on the design of sorption systems. The relation between cesium adsorption and time was performed by several kinetic models to clarify the rate and mechanism of the adsorption reaction. Ho and McKay model (pseudo-second-order model) assumes that the sorption process could be a pseudo-chemical reaction process. In which, all adsorption steps including external/internal diffusion and adsorption are lumped together.^26^

3 *t*/*q*_t_ = *t*/*q*_2_ + 1/*k*_2_*q*_2_^2^

Plotting of *t*/*q*_t_*versus t* (Fig. 4c) gave a straight line with a slope and an intercept, from which the *q*_2_ (theoretical capacity) and *k*_2_ (rate constant) were calculated. Clearly, a satisfactory harmonization was obtained between calculated and experimental values of *q*_e_ (68.49 mg g^−1^) and gives higher correlation value (0.97183) than that of the first order model, so the Cs(i) ions sorption rate on the MMT-IEX could be modeled with the second order model. In general, the sorption process is known to proceed through the following steps; bulk diffusion, film diffusion, pore diffusion and chemical reaction. The rate-limiting step results from one of the above steps or usually from the combination of them. For gaining insight into the sorption kinetic, the datasets time effect was used in the modeling computations and the obtained parameters are discussed in details. McKay model assumes that the adsorption process is controlled by the film- and particle diffusion. During the transport of the solute species from the bulk liquid phase towards the solid adsorbent surface, the boundary layer may play a significant role in the adsorption process. This may be verified by applying the adsorption time data to the liquid film diffusion model:

4 log(1 − *F*) = −*K*_F_/2.303*t*

where *K*_F_ is the film diffusion rate constant (min^−1^) and *F* equals the value of *q*_t_/*q*_e_. In case the plot of log(1 − *F*) *versus t* gives a straight line with intercept almost zero, this indicates that the adsorption may be controlled by the diffusion through the liquid film at the MMT-IEX interface (Fig. 4d). The rate constant for liquid film diffusion (*K*_F_) was determined to be 0.028. The non-zero intercept indicates that the film diffusion is not the rate-determining step in the studied adsorption process. The time dependence data of cesium adsorption by the mesoporous MMT-IEX was modeled by Weber–Morris model to clearly know whether intra-particle diffusion or film diffusion is the rate-limiting step.^27^ This model state that the intraparticle diffusivity is constant and the direction of the diffusion process is radial;

5 *q*_e_ = *X* + *K*_i_*t*^1/2^

where *K*_i_ (mg g^−1^ min^−0.5^) and *X* are the intera-particle diffusion rate constant and constant proportional to the boundary layer thickness, respectively. The values of *K*_i_ and *X* were determined from the slope and intercept of the plot *q*_t_*vs. t*^1/2^ (Fig. 4e). Given the linear nature of the plot and the higher correlation value, the adsorption process is obviously controlled by an intra-particle diffusion mechanism. The small value of *X* (0.09) indicates that the boundary layer thickness has a negligible effect on the adsorption process. Bangham model is also applicable to the adsorption systems in which the intraparticle diffusion is the rate-determining step. This model assumes that the diffusion of cesium into the mesopores of MMT-IEX nanoparticle is the rate-controlling step, the adsorption data were applied to Bangham model;^28^

6 log(*C*_i_/*C*_i_ − *Aq*_t_) = log log(*AK*_B_/2.303V) + *B* log *t*

where *B* and *K*_B_ (mL g^−1^ L^−1^) are Bangham constants and *A* is the weight of MMT-IEX per liter of cesium solution (g L^−1^), the straight line with higher correlation value of Bangham model (Fig. 4f) confirms the probability of a pore diffusion controlled adsorption process. As a conclusion of the kinetic studies, it was clear that the Ho and McKay, Weber–Morris and Bangham models are better to describe the adsorption process of cesium ions by the MMT-IEX nanoparticles than the other studied models, and this was supported by the statistical indices obtained for each model.

#### 3.2.3. Effect of Cs(i) ions concentration

**Fig. 5:**
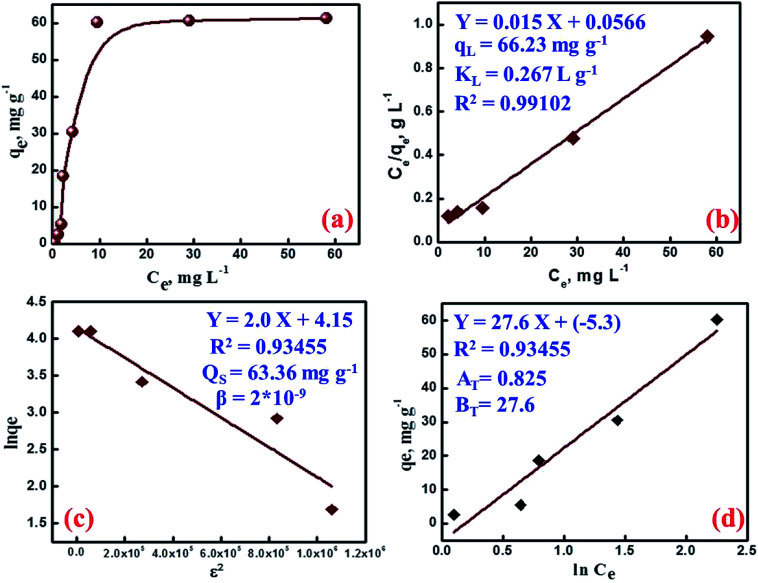
(a) Isotherm of Cs(i) ions adsorption on MMT-IEX from a single ion solution; pH 9.5, 30 mg MMT-IEX weight, 20 mL solution volume, 20 min contact time and 25 °C. (b) Langmuir, (c) D–R, and (d) Temkin plots for the adsorption cesium on the MMT-IEX sorbent.

Studying the effect of initial cesium ion concentration is important to understand and describe the mass transfer resistance of cesium ions between the aqueous and the MMT-IEX phases. The effect of Cs(i) ion concentrations on the adsorption process was investigated within the concentrations range from 0.5 to 150 mg L^−1^. The experimental conditions were set as follows: contact time of 90 min and an adsorbent dose of 1.5 g L^−1^. As clearly shown in Fig. 5a, the uptake of Cs(i) ions by MMT-IEX increases at low metal ion concentration till reaches a plateau at higher concentrations. This behavior is closely related to the concentration of surface active sites available for adsorption. At the beginning of adsorption many active sites would be available for interaction with Cs(i) ions and consequence the active sites become saturated and a dynamic equilibrium is achieved where the adsorption levels off at a maximum adsorption capacity of 60.33 mg g^−1^. A comparative study in terms of adsorption capacity has been carried out with other reported adsorbents, and the results are summarized in Table 1.^2,8,13,14,17,29–36^ Cesium ions may be adsorbed from aqueous media onto the surface of MMT-IEX by several interaction modes. The effective mechanism is related to the nature of adsorption sites, surface properties, affinities of the MMT-IEX adsorbent and the adsorption conditions. The experimental results were treated according to different adsorption models to verify the adsorption parameters that drive the adsorption process. Therefore, the experimental data of cesium adsorption onto mesoporous MMT-IEX were applied to the linear form of Langmuir isotherm:^37^

7 *C*_e_/*q*_e_ = *C*_e_/*q*_L_ + 1/*K*_L_*q*_L_

where *K*_L_ (L mg^−1^) and *q*_L_ (mg g^−1^) are the Langmuir constant and the theoretical adsorption capacity calculated by plotting of *C*_e_/*q*_e_ against *C*_e_. Fig. 5b shows a good straight line, with *R*^2^ = 0.99102, from which the values of *K*_L_ (0.267 L g ^−1^) and *q*_L_ (66.23 mg L^−1^) were determined. The finding that the calculated values of *q*_L_ are comparable to that the experimental values indicate that the adsorption of Cs(i) ions on MMT-IEX is well fitted with Langmuir model. The value of *R*_L_, (*R*_L_ = 1/(1 + *K*_L_ × *C*_i_)), gives an indication for the possibility of the adsorption process to proceed, *R*_L_ > 1.0 unsuitable; *R*_L_ = 1 linear; 0 < *R*_L_ < 1 suitable; *R*_L_ = 0 irreversible. The value of *R*_L_ was found to lie between 0.043 and 0.931 indicating the suitability of the MMT-IEX as an adsorbent for the recovery of Cs(i) ions.

**Table 1:** Comparison of the adsorption capacities of Cs(i) ions onto various absorbents

Adsorbent	Uptake, mg g^−1^	Ref.
Silica/dibenzo-18-crown-6 ether	27.40	2
Silica/benzo-*p*-xylyl-22-crown-6-ether	86.28	8
MCM-41/zinc hexacyanoferrate	103.09	13
Silica/copper(ii) ferrocyanide	17.10	14
Titanium oxide	120.00	17
NH_4_HPA/SBA-15	70.90	29
Vermiculite/ethyl amine	56.92	30
K_2_[CoFe(CN)_6_]	141.44	31
Iron ferrite	155.00	32
Polyaniline/titanotungstate	217.00	33
Silica/calix[4]arene-crown	30.17	34
Zirconium oxide	40.00	35
Hydroxyapatite	69.49	36
MMT-IEX	60.33	This work

The Dubinin–Radushkevich (D–R) model was applied to estimate the energy of adsorption and nature of the adsorption process. The liner form of this model is more general than Langmuir model because it considers a heterogeneous surface. The linear form of the D–R isotherm equations is as follow:^38^

8 ln(*q*_e_) = ln *q*_s_ − *β*(*RT* ln(1 + 1/*C*_e_))^2^ = ln *q*_s_ − *βε*^2^

where *q*_s_, and *E* (
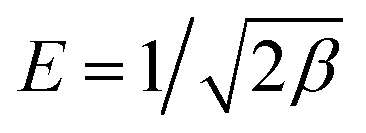
) are the D–R adsorption capacity and the mean free energy, respectively. D–R plot shows a straight line from which the values of D–R parameters were calculated (Fig. 5c). The obtained values of *E* and *q*_s_ are 15.81 kJ mol^−1^ and 63.36 mg g^−1^, respectively. These values agree well with the experimental data indicating a chemical adsorption process. Moreover, the obtained *E* values between 8 and 16 kJ mol^−1^ indicate cation ion-exchange and particle diffusion processes. Finally, Temkin adsorption isotherm considers that heat of adsorption of all active sites decreases linearly with increase coverage due to adsorbent–adsorbate interaction, and adsorption is characterized by a uniform distribution of the binding energies, up to certain maximum binding energy:^28^

9 *q*_e_ = *B*_T_ ln *A*_T_ + *B*_T_ ln *C*_e_

where *A*_T_ (L g^−1^) and *B*_T_ (kJ mol^−1^) are Temkin constants and the value of *B*_T_, (*B*_T_ = *RT*/*b*_T_), is related to the adsorption energy constant (*b*_T_). *A*_T_ and *B*_T_ values were calculated from the slope and intercept of Temkin plots (Fig. 5d). The estimated *B*_T_ values of MMT-IEX were found to be 27.614 kJ mol^−1^ (>40 kJ mol^−1^), confirming that the adsorption process of Cs(i) on MTT-IEX proceed through ion exchange mechanism.

## 4. Selectivity assessment

**Fig. 6:**
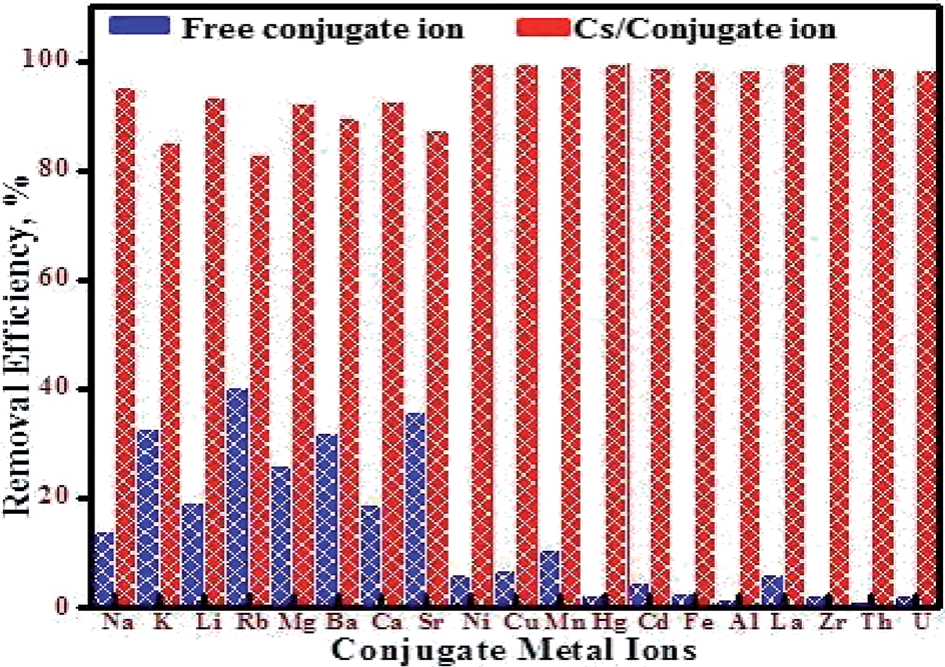
Selectivity profiles of mesoporous MMT-IEX towards Cs(i) ions during the addition of various foreign metal ions (5 mg L^−1^) at optimal adsorption conditions (MMT-IEX weight 40 mg, volume solution 30 mL, eq. time 90 min and 25 °C). The interfered cations listed in the order (1–20) Li(i), Na(i), K(i), Mg(ii), Ca(ii), Sr(ii), Ba(ii), Rb(i), Fe(iii), Al(iii), Ni(ii), Cu(ii), Hg(ii), Pb(ii), Mn(ii), Cd(ii), La(iii), Zr(iv), U(vi) and Th(iv).

The specificity of the fabricated MMT-IEX adsorbent was examined by evaluating its capability to selectively remove radioactive cesium ions, especially from its co-exist ions present in seawater and aquatic nuclear waste. The effect of competing species like Li(i), Na(i), K(i), Mg(ii), Ca(ii), Sr(ii), Ba(ii), Rb(i), Fe(iii), Al(iii), Ni(ii), Cu(ii), Hg(ii), Pb(ii), Mn(ii), Cd(ii), La(iii), Zr(iv), U(vi) and Th(iv) on Cs(i) removal from aqueous solutions onto the mesoporous MMT-IEX was evaluated using three sequence cases at its optimal experimental conditions of pH 9, MMT-IEX amount 40 mg, solution volume 30 mL at 25 °C. Firstly, in binary solution, the interfering ions (5 mg L^−1^) were initially added to 1 mg L^−1^ of Cs(i) ions. Result of selectivity studies from this binary system indicates that the metal ions such as K(i), Rb(i) and Sr(ii) ions exerted a higher effect on the removal of Cs(i) ions than other ions (Fig. 6). Secondly, in the multi-mixture system, the effect of the interfering ions on Cs(i) removal was examined by carrying out the selectivity experiments in 4 groups of mixtures and each group contains 6 conjugated metal ions. The composition of each group of ions mixture includes, Group-I: Cs(i), Li(i), K(i), Na(i), Rb(i) and Sr(ii), Group-II Cs(i), Mg(ii), Ca(ii), Ba(ii), Fe(iii) and Cd(ii), Group-III: Cs(i), Al(iii), Ni(ii), Pb(ii), Mn(ii) and Hg(ii), Group-IV: Cs(i), Cu(ii), La(iii), Zr(iv), U(vi) and Th(iv). In this removal system the metal ions of Group-I displayed a greater impact on removal efficiency (%) of Cs(i) ions than other groups (namely, hydrated ion size and relative charge density) at pH 9. Therefore, the removal efficiency of Cs(i) ions was increased gradually from Group-I to Group-IV; 88.4 < 93.1< 95.5 < 98.6%, respectively. Finally, the removal applicability of Cs(i) from simulated wastewater sample was carried out by initially adding 5 mg L^−1^ of Li(i), Na(i), K(i), Mg(ii), Ca(ii), Sr(ii), Ba(ii), Rb(i), Fe(iii), Al(iii), Ni(ii), Cu(ii), Hg(ii), Pb(ii), Mn(ii), Cd(ii), La(iii), Zr(iv), U(vi) and Th(iv) ions to 1 mg L^−1^ Cs(i) ion. The sample solution containing cesium (pH 9) was studied according to the ion-selective procedure at optimal experimental conditions. Our findings that MMT-IEX achieves a high-performance recovery of 91.77% for Cs(i) ions. The selectivity of MMT-IEX can be ascribed to its high binding affinity for the interfering metal ions due to the intrinsic mobility of Cs(i) ions to bind MMT-IEX through ion exchange process derived from hydrated ion size and relative charge density (*i.e.* smaller the size of ion, the more highly is it hydrated). Greater the mass of a hydrated ion, the lower is its ionic mobility, which indicates that K(i) and Rb(i) can occupy a hydrated state more easily instead of adsorption on MMT-IEX because of its higher hydration energy.^39^ Moreover, the ICP-OES analysis of the collected Cs(i) ions solution indicates that approximately 99.3% of the Cs(i) metal ions were released using 0.05 M HNO_3_.

## 5. Conclusions

A nanostructured mesoporous calcium hydrogen phosphate (MMT-IEX) was fabricated through sol–gel method. The MMT-IEX was characterized using FT-IR, XRD, SEM, TG, HRTEM, DLS and N_2_ adsorption/desorption techniques and the results confirmed the adsorbent formation as nanostructured mesoporous calcium phosphate. MMT-IEX was used as an effective sorbent for cesium ions from their aqueous solutions. The adsorption kinetic data was best modeled by Ho and McKay model. The adsorption isotherm data was best modeled by Langmuir, Temkin and D–R isotherm. All the experimented data are discussed revealing the success of MMT-IEX as cation exchanger for the adsorption and separation of Cs from their media to a large extent. In summary, the fabricated monetite meso-sorbent achieves high-performance collection, recovery, and extraction of Cs(i) ions. Therefore, it is highly applicable to the environmental cleanup of radioactive cesium.

## Conflicts of interest

There are no conflicts to declare.

## Supplementary Material
